# The Medicalization of Love

**DOI:** 10.1017/S0963180114000206

**Published:** 2015-07

**Authors:** BRIAN D. EARP, ANDERS SANDBERG, JULIAN SAVULESCU

**Keywords:** medicalization, love, love drugs, neuroenhancement, marriage, ethics

## Abstract

Pharmaceuticals or other emerging technologies could be used to enhance (or diminish) feelings of lust, attraction, and attachment in adult romantic partnerships. Although such interventions could conceivably be used to promote individual (and couple) well-being, their widespread development and/or adoption might lead to the ‘medicalization’ of human love and heartache—for some, a source of a serious concern. In this essay, we argue that the medicalization of love need not necessarily be problematic, on balance, but could plausibly be expected to have either good or bad consequences depending upon how it unfolds. By anticipating some of the specific ways in which these technologies could yield unwanted outcomes, bioethicists and others can help to direct the course of love’s medicalization—should it happen to occur—more toward the ‘good’ side than the ‘bad.’

. . . Do not all charms fly

At the mere touch of cold philosophy?

There was an awful rainbow once in heaven:

We know her woof, her texture; she is given

In the dull catalogue of common things.

Philosophy will clip an Angel’s wings,

Conquer all mysteries by rule and line,

Empty the haunted air, and gnomed mine—

Unweave a rainbow, as it erewhile made

The tender-person’d Lamia melt into a shade.

John Keats, *Lamia* (1820)

## Introduction

Scientists have begun to unravel some of the neurochemical and other brain-level factors that underlie human lust, attraction, and attachment—on some accounts, the biological building blocks of love.^1^ The neurobiologist Larry Young, for example, has characterized romantic love as “an emergent property of a cocktail of ancient neuropeptides and neurotransmitters,” leading him to speculate that drugs “that manipulate [our] brain systems at whim to enhance or diminish our love for one another may not be far away.”^2^ More recent research suggests that such drugs—at least in a nascent form—may already be partly available,^3,4^ and future versions may be even more potent.^5^ Granting, then, that pharmaceuticals and other emerging technologies could be used to “enhance” or “diminish” our love-related drives, emotions, and attachments, could such manipulations ever be justified?

Over a series of recent papers, we have argued that they could—at least for some individuals and some couples, under certain types of conditions.^6,7,8,9,10^ Although several commentators agreed with our reasoning concerning the specific set of cases we explored^11,12,13,14,15^—including both enhancing and diminishing interventions^16^—some also cautioned that the *wider social consequences* of developing love-affecting technologies would have to be considered as well.^17,18,19,20^ Some of these potential consequences have been addressed elsewhere,^21^ but others remain to be analyzed. In the present article, we consider an objection that has not yet received significant attention, namely that the development or the use of such technologies would lead to the ‘medicalization’ of human love and heartache.

Before we turn to this objection, we need to describe what kind of technology we have in mind for this analysis. More thorough discussions of the neuroscience involved can be found in earlier papers,^22,23,24,25^ but the basic principle is as follows. Underlying human romantic attachment is a collection of interlocking brain systems that are hypothesized to have evolved to suit the reproductive needs of our ancestors. Brain chemicals such as oxytocin, dopamine, testosterone, and many others seem to regulate our interpersonal drives and emotions, including the formation of romantic pair-bonds. Although love is not simply reducible to these brain chemicals or pathways—and although there are many different theories of love, including how it should be defined—what is clear by now is that these underlying phenomena do much to shape (as well as to respond to) our higher-level romantic experiences, across a wide range of theoretical conceptions.

What scientists are now beginning to discover is that these brain systems can be influenced, certainly in animal models, and at least to some degree in humans as well, by administering or blocking the relevant chemical compounds. For example, by administering a dose of oxytocin—by injecting it directly into the brain—scientists can induce a pair-bond in a certain species of vole, even if the voles have not yet engaged in any mating behavior.^26^ They can also reverse this process, by administering an oxytocin blocker: voles that would otherwise have formed a monogamous attachment fail to do so and express interest in novel sexual partners.^27^ Similarly, in humans, synthetic oxytocin can be administered as a simple nasal spray (and seems to strengthen attachment-related representations as well as romantic bonding cues),^28^ while the modulation of other neurochemicals can interfere with relationship attachments, for example, by diminishing the sex drive.^29^ The question raised by these kinds of findings is whether we could (or should) attempt to harness such information in order to pursue a research program into the neuromodulation of human love and relationships.

## The Objection

One reason to think that we should not pursue such a program is that it might lead to love’s medicalization. ‘Medicalization’ has been defined by Joseph E. Davis as “the process by which medical definitions and practices are applied to behaviors, psychological phenomena, and somatic experiences not previously within the conceptual or therapeutic scope of medicine.”^30^ The concern that such a process might spread to the case of love has been raised by the sociologist John Evans:

[Many people have] reached the normative conclusion that they do not want to live in a world where increasing swaths of human experience are under the logic of medicine. There are, or should be, experiences that use an older logic, which are under the jurisdiction of another profession or under no jurisdiction at all. *We can all fear the medicalization of love*.^31^

Evans portrays love as an outer limit for the process of medicalization—a bright red line that simply should not be crossed. In other words, although we might potentially have to tolerate, if not condone, such phenomena as the widespread use of Prozac for the treatment of depression^32^ (the “medicalization of misery” according to some^33^), or even the “invention” of ADHD^34^ to justify the administration of Ritalin in obstreperous young boys (see Sami Timimi’s discussion of the “medicalization of childhood”^35^), critics of medicalization might suggest that we should *not* be prepared to accept the encroachment of medicine into matters of the heart. As Eric Parens notes, “At work in Evans’s claim is the at-first seemingly obvious assumption that medicalizing love is bad, full-stop.”^36^ No further argument needed.

Our aim in this article—or one of our aims—is to challenge this basic assumption. As a point of departure (although this will not be the main thrust of our argument), we note that there exists a substantial amount of evidence that human love and relationships are *already* deeply implicated in such uncontroversially ‘medical’ phenomena as physical health and longevity.^37^ Positive interpersonal relationships yield a wide array of medical benefits including improved coping with major illnesses^38^; indeed, the “influence of social relationships on the risk of death [is] comparable with [or even exceeds] well-established risk factors for mortality” such as smoking, drinking, lack of exercise, and obesity.^39^ By contrast, relationship dysfunction and loneliness are damaging to health and well-being and can lead to such outcomes as illness, depression, and inflamed immune responses of the type that contribute to arthritis and coronary heart disease.^40,41^ Hence, as we argued in a recent paper: “If relationship dysfunction . . . turns out to be at the ‘root’ of such serious problems as heart disease or arthritis, then treatment modalities aimed at addressing relationship health in the first instance would seem to be well worth investigating.”^42^

Of course, relationship ‘treatments’—even if warranted—would not need to be biochemically based. Most existing therapies are not.^43^ Perhaps the worry, then, is not about medicalization per se, but rather about *pharmaceuticalization*, a related but distinct phenomenon.^44^ For, as Parens states: “Insofar as relationship difficulties are a normal human problem, and insofar as marriage counseling is sometimes done by people with medical degrees, it seems fair to say that . . . relationship counseling to maintain a marriage relationship could be [considered] a ‘good’ form of medicalization.”^45^ That much seems uncontroversial.

What about the addition of drugs, then? If we are prepared to agree that relationship counseling is, or can be, an acceptable form of medicalization—and that insights gleaned from the ‘scientific’ study of factors that promote, or detract from, relationship health and functioning can reasonably be applied in such settings^46^—then it would seem to be important to determine whether (or to what extent) the additional, adjunctive use of a bond-enhancing neurochemical substance would alter the underlying moral equation. Such a substance would not work to create love ‘magically,’ of course, but it might certainly help it along by acting on the underlying substrates of attachment,^47^ or by promoting more empathic states of mind.^48^ Imagine the following scenario:

John and Lisa are a married couple. They are in a relationship counseling session and are working on their communication. Their counselor, Mary, has shown them research suggesting that the neurotransmitter oxytocin—administered via a nasal spray—can decrease levels of stress while simultaneously increasing productive communication behaviors in some couples.^49^ Mary has explained the risks and benefits of using oxytocin in a well-controlled setting, and John and Lisa have undertaken a prescreening exercise to ensure that neither of them is at serious risk for adverse outcomes. They both agree that they would like to improve their communication; they are convinced, with good reason, that the addition of oxytocin to their counseling regime could be helpful toward that end; and they give their (fully informed) consent to having Mary administer a dose of oxytocin, at regular intervals, under her careful and expert guidance.

Is this a scenario to be feared—even at a larger scale? It is not immediately obvious that it is. It seems, therefore, that further work is needed to bring to the surface some of the hidden considerations that might be buttressing this sort of perspective—namely, that we can (or perhaps that we should) “all fear the medicalization of love.” Peter Conrad has identified a number of worries about medicalization that recur throughout the literature.^50^ These include the following:

- *Worry 1: The Pathologization of Everything.* Medicalization can transform ‘ordinary’ human differences and experiences into ‘pathologies,’ redefining what is “normal, expected, and acceptable in life” through the ever-expanding application of disease categories and labels.^51^
- *Worry 2: The Expansion of Medical Social Control.* Medicalization can expand the scope of medical surveillance and thus medical social control over so-called deviance.^52^ It can also create openings for pharmaceutical companies and other ‘medical entrepreneurs’ to sell us drugs we don’t need for diseases we don’t have (or that have been simply invented out of whole cloth), thereby expanding the power of Big Pharma to meddle in our lives.^53^
- *Worry 3: The Narrow Focus on Individuals Rather Than the Social Context.* Medicalization can lead to the “individualization of social problems,” taking resources and attention away from the wider social and contextual factors that may be creating the need for ‘treatment’ in the first place.^54^ This concern has been summarized by Barbara Wootton: “Always it is easier to put up a clinic than to pull down a slum.”^55^

A related worry can be discerned in the work of John Bancroft,^56,57^ Karen Houppert,^58^ Leonore Tiefer,^59^ Mark Rapley et al.,^60^ and many others and can be expressed as follows:

- *Worry 4: The Narrow Focus on the Biological (or Neurochemical) Rather Than the Psychological.* Medicalization can lead to a sort of bio-reductionism, favoring molecular-level understandings of phenomena that are in reality much more complex. Such reductionism can lead to an undue emphasis on molecular-level interventions as well, promoting a “view of ourselves as objects that can be fixed [rather than] as subjects who can be influenced by reasons.”^61^

A final worry that is sometimes raised in the context of concerns about medicalization—and one that follows naturally from Worry 4—is that drug-based treatments (in particular) have the potential to render *inauthentic* the feelings or behavior of the individuals undergoing the intervention, thereby undermining their ‘true’ selves. Hence:

- *Worry 5: The Threat to Authenticity and the Undermining of the True Self.* Medicalization—and pharmaceuticalization more specifically—might serve to separate the individual from un-drug-mediated experiences and hence from how they “really are” and how “the world really is.”^62^

What these worries seem to indicate is that medicalization—and pharmaceuticalization—have the potential to lead to a number of potentially bad consequences. But they can have good consequences as well. The medicalization of abortion, for instance, which many regard as a positive development, was responsible for transforming the “ending [of] unwanted pregnancy . . . from a criminal act to a medical one” even though “pregnancy . . . is not a disease.”^63^ There are numerous additional examples of medicalization that one might think have been on balance beneficial, such as the medicalization of epilepsy (formerly a ‘spiritual’ condition), the medicalization of alcohol addiction (formerly ‘deviant drinking’), or the medicalization—and hence treatment—of pain. Parens observes that a “blanket condemnation” of medicalization would fail “to acknowledge the respect[s] in which women (and men) use medical technologies to gain control over their lives to promote their own flourishing.”^64^ In other words: there are “good and bad forms of medicalization.”

Following on from this view, as Matthis Synofzik has forcefully argued,^65^ questions about the prudent, legitimate use of psychopharmacological treatments and other forms of medical intervention cannot be discussed in purely abstract terms but rather require a domain- and even case-specific analysis. We agree and would like to attempt such an analysis for the case of love, beginning with a discussion of the first two worries introduced above.

## Worries 1 and 2: The Pathologization of Everything and the Expansion of Medical Social Control

How might these specific worries apply to the medicalization of love and romantic relationships? One way to begin to understand the concern is by recalling the history of medicalization as applied to *sex* and *sexual attraction*: obviously love, sex, and relationships are intimately intertwined.^66^ As numerous critics have noted, the subjugation of ‘natural’ sexual variation to the disease labels of medicine has led to numerous problematic outcomes. Same-sex attraction, for example, was regarded as a medical problem by the mainstream profession of mental health as recently as the 1970s, and misguided attempts at ‘treatment’ and ‘conversion therapy’ are still being carried out in some conservative religious communities to this day.^67^ Indeed, there are countless other examples of ordinary sexual and relational differences being overtly and harmfully pathologized throughout the course of prior centuries.^68^

Kristina Gupta has recently warned that restrictive norms about monogamy might lead to stigmatization of nonmonogamous relationships, driving some individuals to turn to neurotechnological interventions to try to conform to prevailing expectations.^69^ One could even imagine the invention of various relationship ‘diseases’—perhaps promoted by pharmaceutical companies in their hungry pursuit of profit—ranging from ‘commitment phobia’ to ‘adultery proneness syndrome’ to ‘hypoactive love disorder.’ One related example from real life is the conspicuous invention of “hypoactive sexual desire disorder,”^70^ an instance of crude neuroreductionism (see our paper “Neuroreductionism about Sex and Love” for further discussion).^71,72^

We agree that these are outcomes to be feared—or at least regarded with serious suspicion. But several points of nuance need to be raised. First, as Synofzik has pointed out, the disease-oriented focus of medicine seems to be diminishing, rather than increasing, over time. The older model, which centered on “objective clinical-pathologic indices and favored a paternalistic physician-patient relationship” is shifting more toward “a new quality of life-oriented focus which emphasizes a patient’s subjective well-being and individual life preferences within a coequal physician-patient relationship.”^73^ In addition, the attempts of pharmaceutical companies and the medical-industrial complex to conjure diseases out of thin air is being met with increasing public skepticism and awareness.^74,75^

This does not mean that opportunities for abuse do not persist. But it does show that the problematic consequences associated with medicalization—including rampant pathologization of natural differences (whose ‘harmfulness’ may be socially constructed and/or based upon flawed moral thinking)—are not unavoidable and need not be left unchecked. Davis, for example, highlights the growing leverage of social movements, grassroots efforts, and patient advocacy groups that work “to secure medical recognition for . . . condition[s] or [diagnoses]” that are consistent with their own norms and values—as well as *de*medicalization of those that are inconsistent with those values (as illustrated by the case of homosexuality)—even in the face of medical resistance.^76^ In addition, Gupta has outlined several measures that could be put in place to diminish the restrictive normalizing pressures of any future technologies, including passing certain restrictions on the activities of drug companies and making changes to the curricula of medical education programs.^77^

Finally, ethicists, including Synofzik, are increasingly arguing for the permissibility—even prudence—of allowing the administration of psychotropic substances on the basis of their ability to improve individuals’ quality of life—even if there is no particular ‘illness’ to diagnose (in the sense of a mechanical breakdown of some brain system, for example). This would further defuse the potential problem of the pathologization of everything because it would separate *treatment* (applying a medical technology) from *pathology* (identifying a ‘real’ disease). Such a paradigm would obviously blur the distinction between treatment and enhancement (or else count the former as a subclass of the latter), a topic of much debate in the recent literature. For enhancement theorists such as ourselves—who argue that the goal in either case should be to improve well-being—this would be a welcome shift.^78^ Thus, as we argued recently with respect to romantic relationships, “treatment [paradigms] should hinge on considerations of harm and well-being, rather than on definitions of disease.”^79^

Taking these considerations together, it seems not only that the old Foucauldian specter of pervasive medical surveillance—of oppressive normalization and top-down control—may be losing considerable steam, but also that, with conscious and deliberate effort, this trend could be reinforced.^80^ The take-away lesson here is that, as long as one does not subscribe to a strong technological determinism, according to which the mere availability of a given technology inevitably produces certain social outcomes (thus making most ethical discussion irrelevant), the solution does not lie in avoiding potentially problematic technologies altogether. Instead, it has to do with attempting to anticipate any difficulties that may be associated with those technologies and then modifying the context (social, legal, etc.) in which they would most likely be used.

## Worries 3 and 4: The Narrow Focus on Individuals (and Biology) Rather Than on Social Context (and Psychology)

What about this concern? Conrad has expressed it well: “Medicalization can obscure the social forces that influence well-being. For example, by focusing completely on the neurobiological features [of some condition, it may be viewed as a genetic or biological problem], and [thus] treated predominately with [drugs]—while the social environments that . . . feed [the condition] are not altered.”^81^ Unfortunately, this “focus on the individual has reinforced the proclivity of treating complex societal problems with technological fixes . . . rather than by changing the social structure.”^82^

The relevance of this concern to relationships can be illustrated by a story from Jonah Lehrer.^83^ It tells of a psychiatrist who was all too eager to prescribe antidepressants to his patients, failing to “distinguish between suffering rooted in [their] dysfunctional bodies and suffering rooted in their minds or social contexts.”^84^ The punch line comes when he asks one of his patients how her antidepressant medication is working. “It’s working great,” she says. “I feel so much better. But I’m still married to the same alcoholic son of a bitch. It’s just now he’s tolerable.”

Diana Aurenque and Christopher McDougall have made a similar point using a slightly different example. Focusing on a case of domestic abuse in which the victim’s emotional attachment to her abuser is preventing her from leaving the relationship, they argue that “the first and most obviously justified intervention in [this] case is . . . not to drug [the woman] into unfeeling, but to alert the authorities to the violence and refer her to supportive social and legal services.”^85^ Indeed, as Purdy notes, administering ‘medicine’ to women in oppressive social situations may be no better than putting on a superficial Band-Aid and can lead to far worse outcomes overall than would be achieved by changing the conditions in which they live.^86^

These are unquestionably valid observations. But here too there is room for nuance, as Purdy herself points out. “These points imply that women’s health will be far better protected by political action than by medicalization,” she writes. However, “does it follow that women’s health . . . should [therefore] be excluded from the medical realm? Surely not. [For] even in the best [of] circumstances, a few women would require all the help that medicine can offer, and such help needs to be made available to them.”^87^ The key is for societies, including policymakers, to consider medical interventions as complements to social change, rather than as replacements for it. As Gupta writes with respect to love-altering technologies specifically, individual/structural and biological/social factors are co-constitutive:

Interventions aimed at the individual may be effective and may have reverberating effects on the broader social issues, and vice versa. I would simply encourage scholars considering the ethics of biotechnological interventions to address problems with an individual and social component to emphasize the importance of integrating these individual interventions with social interventions and to consider how the two might work in tandem to achieve change. Combined with efforts to address the social factors that contribute to [problematic relationships or forms or states of love] and with measures in place to mitigate the normalizing potential of these interventions, [love-altering] technologies may indeed increase human flourishing.^88^

Finally, we should note that—in general—the ‘treatment’ for some condition need not be determined by its primary etiology. To return to the example of depression, as Synofzik points out: “If we alleviate our depressive mood by going running, sunbathing or eating a piece of chocolate, we improve a predominately psychosocial problem by means of a treatment which is not in a psychosocial, but in a physical and partly even neurochemical modality. . . . Thus the modality difference between etiology and treatment cannot function per se as the normatively decisive factor.”^89^ As we have argued previously, therefore, the modality or the type of intervention does not matter as much from a moral perspective as whether it improves the well-being of the people involved, all things considered.^90^

## Worry 5: The Threat to Authenticity and the Undermining of the True Self

The final concern is that medicalized treatments and/or enhancements of ‘love’ might threaten authenticity or selfhood. The President’s Council on Bioethics has stated this concern as follows: “We might succeed in easing . . . suffering [or ‘improving’ a relationship] at the risk of falsifying our perception of the world and undermining our true identity.”^91^ With respect to bond-enhancing substances in particular, the worry might be that they could make someone more approachable, more trusting, or more empathic than she ‘really is,’ or even more likely to feel love than would otherwise be the case.

We do not underestimate the importance of such concerns. But it is important to think through some of the specifics of what exactly is being implied. First, it might be pointed out that a person can ‘enhance’ her own romantic chemistry behaviorally—by receiving a massage, engaging in sexual intercourse, going on an adventure with her partner, or attending couple’s therapy. All of these activities release such neurotransmitters as oxytocin and dopamine, albeit endogenously and in a particular behavioral context. Yet if someone pursued these activities (sex, massage, etc.) with the explicit aim of improving her relationship—by increasing trust, empathy, and interpersonal connection—it would be difficult to see how her authenticity had been compromised in any obvious way.^92^

That being the case, however, it is not at all clear that the exogenous modification of that same brain chemistry—*especially in a similar behavioral context*, yet through the use of an oxytocin nasal spray, as in the example of John and Lisa—would introduce a special moral problem. Rather, such modification might even promote a person’s ability to live her life ‘authentically’ and in accordance with her goals and values. As Niklas Juth has written, “In general, plans require capacities in order for them to be put [into] effect and [drug-based treatments may] increase our capacities to do the things we need to do in order to effectuate our plans.”^93^ If the administration of certain ‘love drugs’ turns out to be effective in promoting states of mind and behavioral dispositions that are conducive to a healthy relationship, then couples may simply have an additional tool at hand to help them pursue their overriding interpersonal aims.

Another concern about authenticity is that drug-based interventions might introduce psychological or behavioral inconsistencies in the person or couple being enhanced—possibly interfering with the sense that it is the ‘same person’ (or relationship) through time. This does seem like a meaningful worry. However, it is an empirical question whether such inconsistencies would be introduced, and for many neurochemical interventions with which we are already familiar (alcohol, antidepressant medication, etc.), they are not, or do not seem to be, in a reasonably large number of cases. If such inconsistencies were introduced, however, there would still be further questions. To what degree are they introduced? Are they introduced in all people who take such drugs, or only in some people? Can such differences be predicted? Are the inconsistencies—if they have been introduced—necessarily bad? And if they are bad, how do they weigh against the various benefits brought to the relationship or to the individual’s sense of self along other dimensions? In this context, we should remember that even our ‘true’ selves—and experiences/conceptions of love—can be self-contradictory to some extent.

Thus there seem to be two main issues: first, it is unclear whether the administration of love drugs would in fact pose a threat to authenticity (or whether it would do so for some individuals and couples but not others), and, second, it is unclear whether, even if it did pose such a threat, this would entail that it should not be done (under the right kinds of conditions). Indeed, one might wish to consider a more basic issue, which is to ask what exactly the value of authenticity is—and how it weighs against other values. Perhaps it is authentic for a person to be sad and sullen, withdrawn and incommunicative, or cold, unapproachable, and unloving. Yet within the hierarchy of values for a particular couple, a happy or well-functioning relationship may be more important than an abstract notion of authenticity. It would seem reasonable to argue that couples should be able to pursue their highest values by whatever (legal) means they themselves, perhaps in conjunction with the guidance of a trained professional, deem to be most appropriate and most effective.^94^

## Conclusion: Unweaving the Rainbow?

Let us summarize our argument thus far. We have tried to show that the medicalization of love cannot be productively analyzed in abstract terms, because medicalization in general can be either good or bad, and which one it is, on balance, depends on a number of specific and even idiosyncratic factors. These factors, we have suggested, vary along with the phenomenon (such as love) in question, the individuals or groups involved, and the wider social context. By looking at five particular worries—or classes of worry—that pertain to the medicalization of love and relationships, we have argued that certain bad consequences could reasonably be expected to follow, although they might be softened or even reversed by other factors, including by preemptive ethical deliberation, as well as by the implementation of a number of policy measures, such as those that have been proposed by Gupta. We hope that this has been sufficient at least to cast doubt upon the view that “we can all fear the medicalization of love” (full stop).

If it has been sufficient, then we have accomplished our main task; and we could end the article here. Yet we think that there may be a deeper worry associated with this debate, and that is that the medicalization of love, or even just the study of love from a scientific perspective, will somehow rob it of its value and importance—reducing it to a set of mindless chemicals. As Wordsworth wrote in *The Tables Turned*, perhaps “Our meddling intellect / Mis-shapes the beauteous forms of things.” Perhaps we have to “murder to dissect.”^95^ One is also reminded of Keats, who, according to a famous biologist’s interpretation, “believed that Newton had destroyed all the poetry of the rainbow by reducing it to the prismatic colours.”^96^

This view has a certain appeal. Part of the magic of love, it seems, is that it can be so mysterious and wonderful—it can sweep us off our feet, as though by a force outside ourselves. Do we really want to put it under a microscope? All for the sake of ‘health’ or some abstract notion of ‘well-being’? Perhaps we do not. But one might also want to ask whether the value of love resides in its mysteriousness—in our inability to understand it—or rather more generally in how it acts in our lives. Neither unpredictability nor ignorance alone makes something worthwhile, and even with a perfect medical understanding of love, we would not be able to predict who we would fall in love with or specifically how the relationship would play out.

Moreover, as Erich Fromm has argued,^97^ there may be a hidden danger in the view that love is something that ‘just happens to us’—when we meet the ‘right’ person, for example—rather than something for which we must take personal responsibility, and work on, and try to improve. Many individuals who are delighted to be ‘swept off their feet’ in the early stages of a romantic relationship are just as devastated, later on, when such feelings begin to fade, seemingly outside of their control. They might even (mis) attribute their changing feelings to something wrong in the partner, or in the relationship, and go rushing into a breakup or divorce. But what if the problem is not in their partner or the relationship, but at least partly in their conception of love? What if to love is to practice an art, as Fromm argued, that requires conscious effort and discipline—as well as knowledge, and therefore understanding? What if knowing how love works, in other words, could help us be better at being in love?

As the biologist Richard Dawkins once wrote, in response to Keats’s lament, “Newton’s unweaving of the rainbow led on to spectroscopy, which has proved the key to much of what we know today about the cosmos. And the heart of any poet worthy of the title Romantic could not fail to leap up if he beheld the universe of Einstein, Hubble and Hawking.”^98^ This observation raises an interesting possibility concerning the scientific investigation of human relationships. Could there be a similar lesson for the biochemical unweaving of romantic love as well? Could it open up similar poetic vistas? And here is a quote from the physicist Richard Feynman, whose relevance to this discussion will make itself clear:

I have a friend who’s an artist and has sometimes taken a view which I don’t agree with very well. He’ll hold up a flower and say “look how beautiful it is,” and I’ll agree. Then he says “I as an artist can see how beautiful this is but you as a scientist take this all apart and it becomes a dull thing,” and I think that he’s kind of nutty. First of all, the beauty that he sees is available to other people and to me too, I believe. Although I may not be quite as refined aesthetically as he is . . . I can appreciate the beauty of a flower. At the same time, I see much more about the flower than he sees. I could imagine the cells in there, the complicated actions inside, which also have a beauty. I mean it’s not just beauty at this dimension, at one centimeter; there’s also beauty at smaller dimensions, the inner structure, also the processes. The fact that the colors in the flower evolved in order to attract insects to pollinate it is interesting; it means that insects can see the color. It adds a question: does this aesthetic sense also exist in the lower forms? Why is it aesthetic? All kinds of interesting questions which the science knowledge only adds to the excitement, the mystery and the awe of a flower. It only adds. I don’t understand how it subtracts.^99^

What if medicalizing love made it even *more* beautiful, for some couples, adding new questions and types of experiences for them to explore? Dawkins and Feynman certainly give us food for thought. Although their perspective may seem implausible, or even alienating, to some, to others it may be interesting and exciting. Whatever the case, we can be sure that love drugs will not be for everyone. Their value and their usefulness, and how they alter one’s conceptions (and/or experiences) of love and relationships, are—and we think ought to be—in the eye of each beholder.

## Notes

1. FisherHE, AronA, BrownLL. Romantic love: A mammalian brain system for mate choice. Philosophical Transactions of the Royal Society B: Biological Sciences 2006;361(1476):2173–86.10.1098/rstb.2006.1938PMC176484517118931

2. YoungLJ. Being human: Love: Neuroscience reveals all. Nature 2009;457(7226):148.1912982810.1038/457148a

3. EarpBD, SandbergA, SavulescuJ. Natural selection, childrearing, and the ethics of marriage (and divorce): Building a case for the neuroenhancement of human relationships. Philosophy & Technology 2012;25(4):561–87.2322662710.1007/s13347-012-0081-8PMC3510696

4. EarpBD, WudarczykOA, SandbergA, SavulescuJ. If I could just stop loving you: Anti-love biotechnology and the ethics of a chemical breakup. The American Journal of Bioethics 2013;13(11):3–17.2416117010.1080/15265161.2013.839752PMC3898540

5. LynchZ. Neurotechnology and society (2010–2060). Annals of the New York Academy of Sciences 2004;1013(1):229–33.1519461810.1196/annals.1305.016

6. SavulescuJ, SandbergA. Neuroenhancement of love and marriage: The chemicals between us. Neuroethics 2008;1(1):31–44.

7. EarpBD. Love and other drugs. Philosophy Now 2012;91:14–17.

8. WudarczykOA, EarpBD, GuastellaA, SavulescuJ. Could intranasal oxytocin be used to enhance relationships? Research imperatives, clinical policy, and ethical considerations. Current Opinion in Psychiatry 2013;26(5):474–84.2388059310.1097/YCO.0b013e3283642e10PMC3935449

9. See note 3, Earp et al. 2012.

10. See note 4, Earp et al. 2013.

11. GuptaK. Anti-love biotechnologies: Integrating considerations of the social. The American Journal of Bioethics 2013;13(11):18–19.2416117110.1080/15265161.2013.839771

12. EnckG. An error theory of biotechnology and the ethics of chemical breakups: It is the reasons, not the pharmaceuticals, that are important in defending against perilous love. The American Journal of Bioethics 2013;13(11):32–4.2416117810.1080/15265161.2013.839762

13. MarchantGE, StevensYA. Involuntary exposures to love-enhancing or anti-love agents. The American Journal of Bioethics 2013;13(11):26–7.2416117510.1080/15265161.2013.839758

14. GarasicMD. Anti-love biotechnology: Was it not better to have loved and lost than never to have loved at all? The American Journal of Bioethics 2013;13(11):22–3.2416117310.1080/15265161.2013.839770

15. BoeschB. A Thomistic account of anti-love biotechnology. The American Journal of Bioethics 2013;13(11):30–1.2416117710.1080/15265161.2013.839753

16. EarpBD, SandbergA, KahaneG, SavulescuJ. When is diminishment a form of enhancement? Rethinking the “enhancement debates” in biomedical ethics. Frontiers in Systems Neuroscience 2014;8(12):1–8.2455079210.3389/fnsys.2014.00012PMC3912453

17. See note 11, Gupta 2013.

18. EprightMC, WallerS. When love hurts children: Controlling the feelings of minors. The American Journal of Bioethics 2013;13(11):28–9.2416117610.1080/15265161.2013.839754

19. WahlertL. Anti-love or anti-“lifestyle”: Historical reflections on reparative therapies for homosexuality. The American Journal of Bioethics 2013;13(11):36–8.2416118010.1080/15265161.2013.840869

20. McArthurN. The heart outright: A comment on “If I could just stop loving you.” The American Journal of Bioethics 2013;13(11):24–5.2416117410.1080/15265161.2013.839773

21. EarpBD, SandbergA, SavulescuJ. Brave new love: The threat of high-tech “conversion” therapy and the bio-oppression of sexual minorities. AJOB Neuroscience 2014;5(1):4–12.2458796210.1080/21507740.2013.863242PMC3932804

22. See note 6, Savulescu, Sandberg 2008.

23. See note 3, Earp et al. 2012.

24. See note 4, Earp et al. 2013.

25. See note 8, Wudarczyk et al. 2013.

26. InselTR, YoungLJ. The neurobiology of attachment. Nature Reviews Neuroscience 2001;2(2):129–36.1125299210.1038/35053579

27. See note 26, Insel, Young 2001.

28. See note 8, Wudarczyk et al. 2013.

29. See note 4, Earp et al. 2013.

30. Davis JE. Medicalization, social control, and the relief of suffering. In: Cockerham WC, ed. *The New Blackwell Companion to Medical Sociology*. Singapore: Wiley-Blackwell; 2010:211–41.

31. Quoted in ParensE. On good and bad forms of medicalization. Bioethics 2013;27(1):28–35, at 33. Note that the paper by Evans has not yet been published, so we must rely on Erik Parens’s reading of the passage. However, we wish to point out that, as quoted, it is unclear whether the claim that “we can all fear the medicalization of love” is one that Evans himself would endorse, or whether, as a sociologist, he is intending only to illustrate the sort of complaint that might be raised by critics of medicalization generally. Thus, our argument should be interpreted as being a response to *the claim* (and the sorts of worries that we think might be lying behind it), rather than as a rebuttal to Evans.2153506210.1111/j.1467-8519.2011.01885.x

32. Kramer PD. *Listening to Prozac*. New York: Viking Press; 1993, at 1249.

33. Rapley M, Moncrieff J, Dillon J, eds. *De-medicalizing Misery*. Great Britain: Palgrave Macmillan; 2011.

34. Baughman Jr FA. *The ADHD fraud: How Psychiatry Makes “Patients” of Normal Children*. Victoria: Trafford; 2006.

36. See note 31, Parens 2013, at 33.

37. Traupmann J, Hatfield E. Love and its effect on mental and physical health. In: Fogel R, Hatfield E, Kiesler S, Shanas E, eds. *Aging: Stability and Change in the Family*. New York: Academic Press; 1981:253–74.

38. PientaAM, HaywardMD, JenkinsKR. Health consequences of marriage for the retirement years. Journal of Family Issues 2000;21:559–86.

39. Holt-LunstadJ, SmithTB, LaytonJB. Social relationships and mortality risk: A meta-analytic review. PLoS Medicine 2010;7(7), e1000316:20.2066865910.1371/journal.pmed.1000316PMC2910600

40. Jaremka LM, Fagundes CP, Peng J. Loneliness promotes inflammation during acute stress. *Psychological Science* 2013;(Early Access):1–9.

41. WilsonC, OswaldAJ. How does marriage affect physical and psychological health? A survey of the longitudinal evidence. IZA Discussion Paper 2005;1619:1–30.

42. See note 8, Wudarczyk et al. 2013, at 482.

43. Though note that MDMA (Ecstasy) was used in the 1980s in couples’ counseling, and that scientific interest in its therapeutic potential has recently been rekindled: Sessa B. Is there a case for MDMA-assisted psychotherapy in the UK? *Journal of Psychopharmacology* 2007;21(2):220–4. There is also anecdotal evidence (Hutchinson, personal correspondence) that MDMA is currently being used, albeit illicitly, by a network of relationship therapists in Canada, although such research cannot be published. Oxytocin is also being used currently by some counselors: Boesveld S. Chemical seduction: How “love drugs” may one day help couples save failing relationships. *National Post* 2013 Mar 30; available at http://news.nationalpost.com/2013/03/30/chemical-seduction-how-love-drugs-may-one-day-help-couples-save-failing-relationships/ (last accessed 19 May 2014).

44. Abraham defines the term “pharmaceuticalization” as “the process by which social, behavioural or bodily conditions are treated or deemed to be in need of treatment, with medical drugs by doctors or patients”: Abraham J. Partial progress: Governing the pharmaceutical industry and the NHS, 1948–2008. *Journal of Health, Politics, Law and Policy* 2009;34:100. See also AbrahamJ. Pharmaceuticalization of society in context: Theoretical, empirical and health dimensions. Sociology 2010;44(4):603–22.

45. See note 31, Parens 2013, at 34.

46. Gottman JM. *Marital Interaction: Experimental Investigations*. Vol. 5. New York: Academic Press; 1979.

47. See note 8, Wudarczyk et al. 2013.

48. Bartz JA, Zaki J, Bolger N, Hollander E, Ludwig NN, Kolevzon A, et al. Oxytocin selectively improves empathic accuracy. *Psychological Science* 2010;21(10):1426–8.

49. DitzenB, SchaerM, GabrielB, BodenmannG, EhlertU, HeinrichsM. Intranasal oxytocin increases positive communication and reduces cortisol levels during couple conflict. Biological Psychiatry 2009;65(9):728–31.1902710110.1016/j.biopsych.2008.10.011

50. Conrad P. *The Medicalization of Society: On the Transformation of Human Conditions into Treatable Disorders*. Baltimore, MD: John Hopkins University Press; 2008.

51. See note 50, Conrad 2008, at 148.

52. ArmstrongD. The rise of surveillance medicine. Sociology of Health & Illness 1995;17(3):393–404.

53. See note 50, Conrad 2008, at 149–51.

54. See note 50, Conrad 2008, at 152.

55. Wootton B. *Social Science and Social Pathology*. New York: Macmillan; 1959.

56. BancroftJ. The medicalization of female sexual dysfunction: The need for caution. Archives of Sexual Behavior 2002;31(5):451–5.1223861410.1023/a:1019800426980

57. BancroftJ. Biological factors in human sexuality. Journal of Sex Research 2002;39(1):15–21.1247625110.1080/00224490209552114

58. Houppert K. *The Curse: Confronting the Last Unmentionable Taboo: Menstruation*. New York: Farrar, Straus and Giroux; 1999.

59. TieferL. The medicalization of sexuality: Conceptual, normative, and professional issues. Annual Review of Sex Research 1996;7(1):252–82.

60. See note 33, Rapley et al. 2011.

61. See note 31, Parens 2013, at 30.

62. Parens E. Toward a more fruitful debate about enhancement. In: Savulescu J, Bostrom N, eds. *Human Enhancement*. Oxford: Oxford University Press; 2009, at 184.

63. PurdyL. Medicalization, medical necessity, and feminist medicine. Bioethics 2001;15(3):256.10.1111/1467-8519.0023511699544

64. See note 31, Parens 2013, at 33.

65. SynofzikM. Ethically justified, clinically applicable criteria for physician decision-making in psychopharmacological enhancement. Neuroethics 2009;2(2):90.

66. It is instructive to note how ‘lovesickness’ emerged as a medicalized condition in the middle ages, with physical causes and treatments: Wack MF. *Lovesickness in the Middle Ages: The* Viaticum *and Its Commentaries*. Philadelphia, PA: University of Pennsylvania Press; 1990. Although this might be a prime example of medical social control, it also shows how mechanistic explanations in terms of mental faculties and the four humors did not lead to a medicalization of everything, despite being seen as explanations for much of human behavior, emotion, and thought.

67. See note 21, Earp et al. 2014.

68. See note 59, Tiefer 1996.

69. Gupta K. Protecting sexual diversity: Rethinking the use of neurotechnological interventions to alter sexuality. *AJOB Neuroscience* 2012;3(3):26.

70. American Psychiatric Association. *Diagnostic and Statistical Manual of Mental Disorders, Fourth Edition (*i*DSM-IV-TR)*. 4th ed. Arlington, VA: American Psychiatric Publishing; 2000.

71. Savulescu J, Earp BD. Neuroreductionism about sex and love. *Think: A Journal of the Royal Institute of Philosophy*; forthcoming.

72. Kirmayer LJ, Gold I. Re-socializing psychiatry. In: Choudhury S, Slaby J, eds. *Critical Neuroscience: A Handbook of the Social and Cultural Contexts of Neuroscience*. Oxford: Wiley-Blackwell; 2012:305–30.

73. See note 65, Synofzik 2009, at 90.

74. Goldacre B. *Bad Pharma: How Drug Companies Mislead Doctors and Harm Patients*. London: Fourth Estate; 2012.

75. ConradP. The shifting engines of medicalization. Journal of Health and Social Behavior 2005;46(1):3–14.1586911710.1177/002214650504600102

76. See note 30, Davis 2010, at 216.

77. See note 69, Gupta 2012.

78. See note 16, Earp et al. 2014, at 4–12.

79. Earp BD, Wudarczyk OA, Foddy B, Savulescu J. Addicted to love: What is love addiction and when should it be treated? *Philosophy, Psychiatry, & Psychology*; forthcoming.

80. See note 30, Davis 2010.

81. See note 50, Conrad 2008, at 152.

82. See note 50, Conrad 2008, at 152.

83. Lehrer J. Depression’s upside. *New York Times Sunday Magazine* 2010 Feb 28:42. Note that this example comes from Parens 2013 (see note 31).

84. See note 31, Parens 2013, at 31.

85. AurenqueD, McDougallCW. Amantes sunt amentes: Pathologizing love and the meaning of suffering. The American Journal of Bioethics 2013;13(11):35.10.1080/15265161.2013.83975924161179

86. See note 63, Purdy 2001, at 255.

87. See note 63, Purdy 2001, at 255.

88. See note 11, Gupta 2013, at 19.

89. See note 65, Synofzik 2009, at 95.

90. Savulescu J, Sandberg A, Kahane G. Well-being and enhancement. In: Savulescu J, ter Meulen R, Kahane G, eds. *Enhancing Human Capacities*. Oxford: Wiley-Blackwell; 2011:3–18.

91. Kass, LR. *Beyond Therapy: Biotechnology and the Pursuit of Happiness*. Washington, DC: President’s Council on Bioethics; 2003, at 227.

92. See note 8, Wudarczyk et al. 2013.

93. Juth N. Enhancement, autonomy, and authenticity. In: Savulescu et al. 2011 (see note 90):36.

94. Another angle on the matter comes from Guy Kahane’s recent essay, “Reasons to Feel, Reasons to Take Pills.” Kahane argues that biochemical modification of certain feelings may be morally permissible if it is the case that one *ought* to feel differently from how one does in fact feel—due, perhaps, to the sometimes irrational nature of human emotional life. Kahane G. Reasons to feel, reasons to take pills. In: Savulescu et al. 2011 (see note 90). Although parent-child love is not the focus of this essay, one argument that has been raised in the literature is that parents *ought* to love their children: Liao S. Parental love pills: Some ethical considerations. *Bioethics* 2011;25(9):489–94. Hence if love drugs could help toward this end, they might be worth considering. On the other side of the equation, romantic passion is also capable of causing people to feel things that they ought *not* to feel. An example we have already discussed is the profound love and attachment an individual might feel for her domestic abuser, preventing her from ending the relationship. If anti-love biotechnology could help break such a dangerous attachment bond, then its use could be permissible in some instances; see note 4, Earp et al. 2013.

95. Wordsworth W. *The Major Works*. Oxford: Oxford University Press; 1798/2000.

96. Dawkins R. *Unweaving the Rainbow: Science, Delusion and the Appetite for Wonder*. New York: Houghton Mifflin Harcourt; 2000, at x.

97. Fromm E. *Art of Loving*. New York: Perennial Classics; 2000.

98. See note 97, Fromm 2000.

99. Feynman R. The pleasure of finding things out. *BBC* [Interview]; 1981; clip available at http://mentalfloss.com/article/32133/richard-feynmans-ode-flower (last accessed 19 May 2014).

